# Assessment of fluid unresponsiveness guided by lung ultrasound in abdominal surgery: a prospective cohort study

**DOI:** 10.1038/s41598-022-05251-6

**Published:** 2022-01-25

**Authors:** Stéphane Bar, Céline Yee, Daniel Lichtenstein, Magali Sellier, Florent Leviel, Osama Abou Arab, Julien Marc, Matthieu Miclo, Hervé Dupont, Emmanuel Lorne

**Affiliations:** 1 Centre Hospitalier Universitaire Amiens-Picardie, Rond-Point du professeur Christian Cabrol, 80000 Amiens, France; 2 Hôpital Ambroise-Paré, Paris, France; 3 Centre Hospitalier de Lens, Lens, France

**Keywords:** Outcomes research, Oedema

## Abstract

A fluid challenge can generate an infraclinical interstitial syndrome that may be detected by the appearance of B-lines by lung ultrasound. Our objective was to evaluate the appearance of B-lines as a diagnostic marker of preload unresponsiveness and postoperative complications in the operating theater. We conducted a prospective, bicentric, observational study. Adult patients undergoing abdominal surgery were included. Stroke volume (SV) was determined before and after a fluid challenge with 250 mL crystalloids (Delta-SV) using esophageal Doppler monitoring. Responders were defined by an increase of Delta-SV > 10% after fluid challenge. B-lines were collected at four bilateral predefined zones (right and left anterior and lateral). Delta-B-line was defined as the number of newly appearing B-lines after a fluid challenge. Postoperative pulmonary complications were prospectively recorded according to European guidelines. In total, 197 patients were analyzed. After a first fluid challenge, 67% of patients were responders and 33% were non-responders. Delta-B-line was significantly higher in non-responders than responders [4 (2–7) *vs* 1 (0–3), p < 0.0001]. Delta-B-line was able to diagnose fluid non-responders with an area under the curve of 0.74 (95% CI 0.67–0.80, p < 0.0001). The best threshold was two B-lines with a sensitivity of 80% and a specificity of 57%. The final Delta-B-line could predict postoperative pulmonary complications with an area under the curve of 0.74 (95% CI 0.67–0.80, p = 0.0004). Delta-B-line of two or more detected in four lung ultrasound zones can be considered to be a marker of preload unresponsiveness after a fluid challenge in abdominal surgery.

The objectives and procedures of the study were registered at Clinicaltrials.gov (NCT03502460; Principal investigator: Stéphane BAR, date of registration: April 18, 2018).

## Introduction

In major surgery, fluid overload has been shown to be associated with increased postoperative complications and length of hospital stay^1,2^. Current guidelines recommend guiding fluid titration by measuring stroke volume (SV) in high-risk surgical patients to obtain an unresponsiveness state, which corresponds to the absence of an increase in SV of more than 10% after a fluid challenge^3,4^. Application of such recommendations has reduced post-operative morbidity and the length of hospitalization.

Based on the study of artifacts over the last 25 years, lung ultrasound has been increasingly used in intensive care units (ICUs)^5^ then emergency rooms^6^, cardiology^7^ and nephrology^8^. The final step of “fluid administration limited by lung sonography-protocol” (FALLS-protocol) consists of the visualization of ultrasound B-lines during fluid therapy^9^. The appearance of B-lines in an area where no B-line was present is diagnosed as a hydrostatic excess of the subpleural interstitial septa. Such appearance of B-lines takes place at an infra-clinical stage^10^. As lung ultrasound is a simple and real-time tool, could it be potentially used in the operative setting to guide fluid titration and avoid fluid overload?

Our main objective was to study the relationship between the appearance of new B-lines (Delta-B-line) and the appearance of fluid unresponsiveness after fluid challenge in the operating theatre. Our secondary objective was to study the relationship between Delta-B-line at the end of surgery and the appearance of postoperative pulmonary complications.

## Methods

### Ethical approval

The objectives and procedures of the study were approved by institutional review board (*Comité de Protection des Personnes Hospital Group Pitié-Salpétrière*, Paris, France; reference PI2017_843_0018) and was registered at Clinicaltrials.gov (NCT03502460; Principal investigator: Stéphane BAR, date of registration: April 18, 2018). The present report adheres to the applicable CONSORT guidelines and was drafted in accordance with the recommendations of the STROBE statement. All patients provided their informed consent prior to inclusion in the study. The study was conducted in accordance with the Declaration of Helsinki on ethical principles for medical research involving human subjects.

This was an observational, prospective and bicentric study performed in the operating theatre of an University Medical Center and a General Hospital Center between May 2018 and October 2019.

### Study population

The inclusion criteria were adult patients undergoing digestive, urological, or gynecological surgery, requiring intraoperative hemodynamic optimization with fluid challenge titration. The exclusion criteria were patients < 18 years of age or under guardianship or curatorship, pregnancy, poor echogenicity due to the presence of an acoustic barrier (pneumothorax, subcutaneous emphysema, etc.), patients with a suspected or proven acute lung disease [pneumonitis, acute respiratory distress syndrome (ARDS)] or chronic interstitial lung disease (chronic interstitial pulmonary disease with fibrosis), emergency surgery or patients who refused to sign the consent form.

### Lung ultrasound

**Figure 1:**
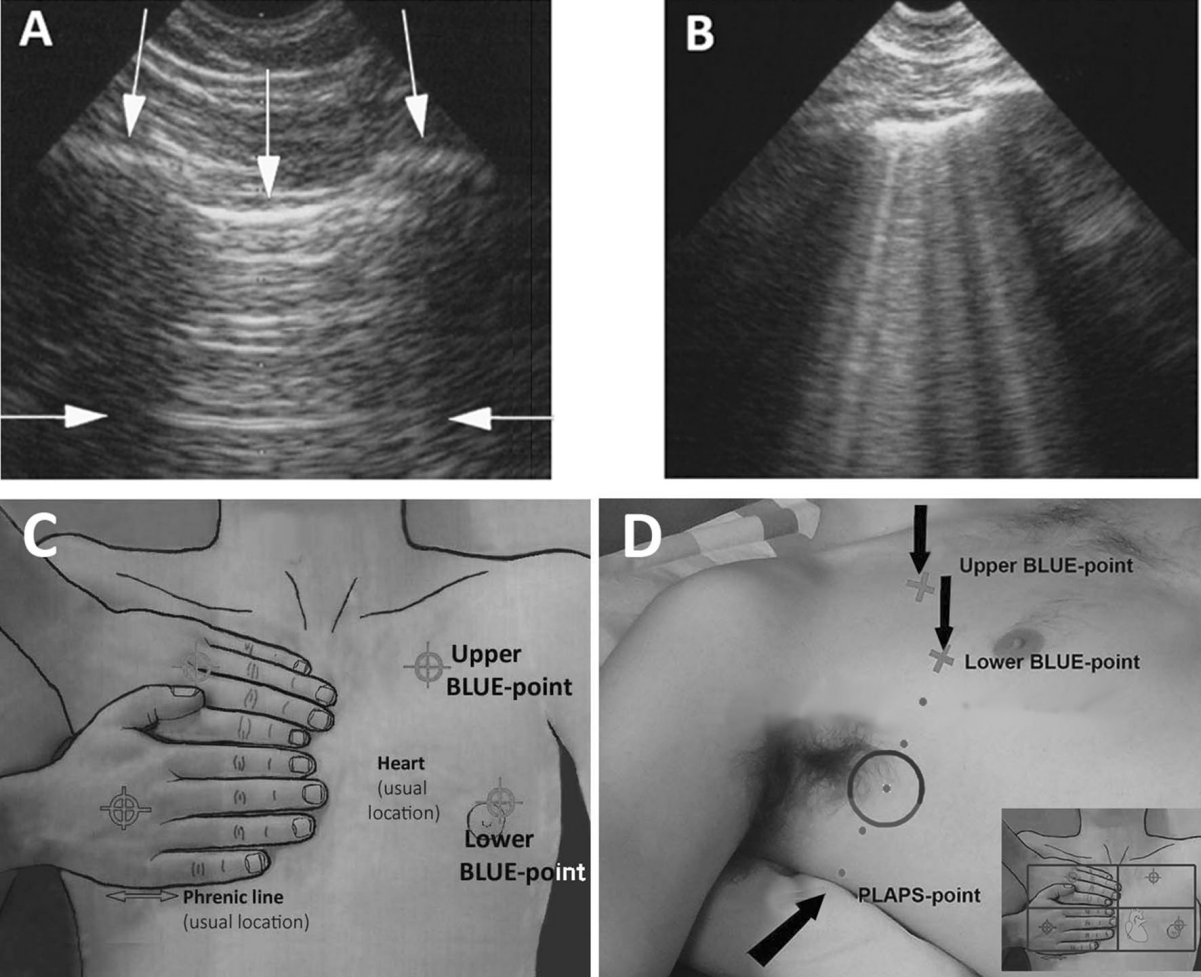
(**A**) Pleural line (vertical arrows indicate the bat sign, with ribs and pleural line). One A-line at the standardized location (horizontal arrows). (**B**) B-lines. 6 B-lines visible between two ribs in short-axis. (**C**) *BLUE-points*. The BLUE-protocol uses three points per lung. Two hands are applied this way, against the clavicule. Two points are anterior, *the upper-BLUE-point* (middle of upper hand, that is, roughly, second intercostal space between parasternal and anterior axillary line) and the lower-BLUE-point (middle of lower palm). One point, continuing transversally the lower BLUE-point as «posterior as possible, is the posterolateral alveolar pleural syndrome-point» (PLAPS-point). Note that the PLAPS-point seems rather cranial, but is in actual fact just a bit above the diaphragm usually. (**D**) *The lateral point*. For adapting the approach to the perioperative setting with its constraints in this study, we took a clinically accessible lateral point located transversally between lower BLUE-point and PLAPS-point, and longitudinally between anterior and posterior axillary line. Note that, if a theoretical point is not accessible for any reason, device or other, the BLUE-points are flexible up to a large tolerance (indicated by the areas in the cartouche).

Lung ultrasound was performed using a Philips iE33 ultrasound device (Philips Medical System, Suresnes, France) and a Philips sectorial, 1 to 5 MHz, wideband S5-1 cardiac probe (Philips Medical System, Suresnes, France). Gain was adjusted to maximize contrast. The A-line and B-line are artifacts recognized by international evidence-based recommendations^11^. We remind that B-line is strictly defined as, always: comet-tail artifact, arising from the pleural line, moving in concert with lung sliding; almost always: long, well defined, erasing A-lines, hyperechoic. In a rib short axis view, three (or more) B-lines are called lung rockets and define interstitial syndrome^12^. Two B-lines are called pre-lung rockets and may indicate a preliminary step of hemodynamic interstitial edema (Fig. 1)^5^.

The zones of analysis were based on previous studies showing that fluid overload is detectable at the anterior chest wall^6^. In order to avoid any collection problem related to surgical drapes, we used an adaptation of the bedside lung ultrasound in emergency-points (BLUE-points), described in Fig. 1^13,14^. Upper and lower BLUE-points are anterior. The lower BLUE-point, when laterally continued, determines a posterior point, the «posterolateral alveolar pleural syndrome-point» (PLAPS-point). In this study, we used a lateral point in between lower BLUE-point and PLAPS-point (roughly between anterior and posterior axillary lines). This lateral point had the advantage of being accessible in spite of the constraints of the operating field. In addition, at the PLAPS-point, B-lines are visible in up to ¼ of healthy subjects^12^.

Number of B-lines were counted bilaterally and was the sum of B-lines at the upper BLUE-points and the lateral points, i.e., four points. The number of B-lines was counted in a rib short-axis scan between two ribs. Care was taken to apply the probe to the same precise area of the same patient during each collection. An alveolar recruitment maneuver was performed two minutes before each lung ultrasound collection (application of a 20 cmH_2_O PEEP for 20 s)^15^. A sufficient time between the end of the maneuver and the data collection in order to avoid any hemodynamic impact of the maneuver on the data (2 min).

Images were stored and the increase in the sum of number of B-lines in the four points (Delta-B-line = number of B-lines after fluid challenge minus number of B-lines at baseline; Final Delta-B-line = number of B-lines at the end of the intervention minus number of B-lines at baseline) was analyzed a posteriori by two lung ultrasound experts blinded to the patient responsiveness status (JM, MM). An examiner was considered to be an expert if s/he had performed at least 50 lung ultrasounds^16^.

### Study protocol

The study began when monitoring of SV was implemented during digestive, urological, and/or gynecological surgery under general anaesthesia. Anaesthesia was induced with propofol and sufentanil or remifentanil and then maintained using target-controlled infusions of propofol and the opioid used for tracheal intubation. Hypnotics and opioids were titrated using the bispectral index (Covidien, Boulder, Colorado, USA), with a target value of between 40 and 60. Neuromuscular blockade was achieved with rocuronium (0.6 mg kg^−1^) or cisatracurium (0.15 mg kg^−1^). All patients were intubated and then ventilated in volume-controlled mode. The tidal volume was adjusted to the ideal body weight (target value: 6 ml kg^−1^ ideal body weight) and the ventilatory rate was adjusted to achieve and maintain an end-tidal CO_2_ pressure of 35 to 37 mmHg. A positive end-expiratory pressure (PEEP) of 5 to 8 cmH_2_O was applied. An alveolar recruitment maneuver was carried out systematically after oro-tracheal intubation^17^.

**Figure 2:**
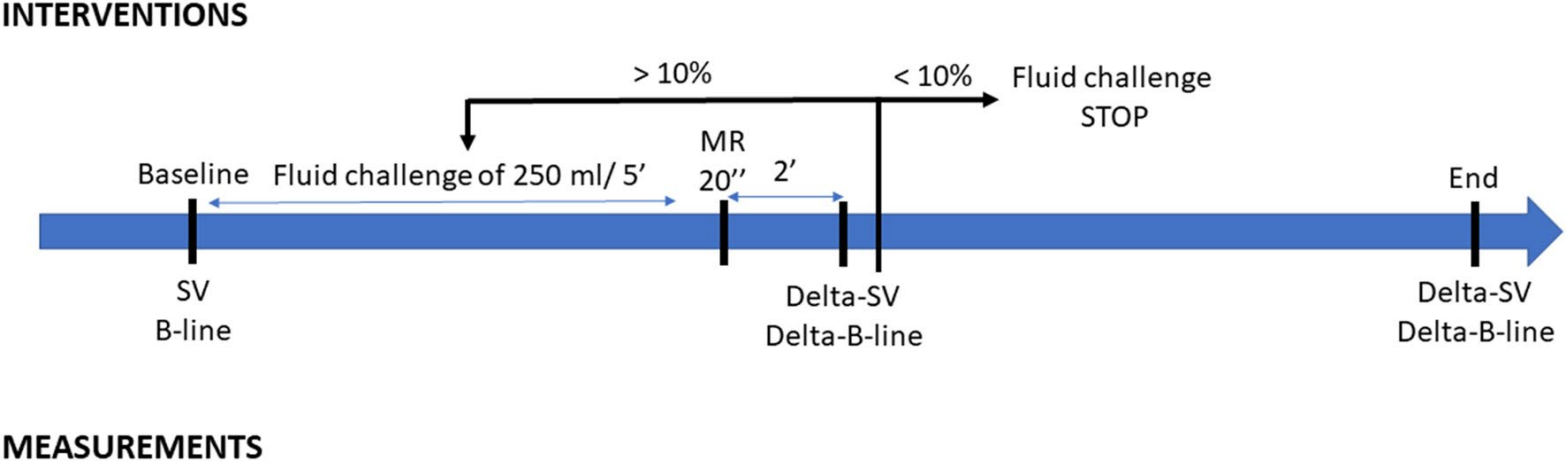
Study protocol.

A DP240 probe was connected to a CardioQ-ODM monitor (Deltex Medical, Chichester, UK) distributed by Gamida. Collection of the SV was averaged over five cardiac cycles. After induction, an initial measurement of the SV and a first lung ultrasound collection in the four zones were performed and stored. Then, SV was measured, and the lung ultrasound collection performed and stored after each fluid challenge with 250 ml of crystalloids which was administered over 5 min^18^. The variation of SV after fluid challenge (Delta-SV) was calculated according to the following formula: Delta-SV (%) = ((SV_after_ − SV_before_)/SV_before_) × 100. A patient was defined as a responder if the Delta-SV was more than 10% after fluid challenge. Fluid challenge was continued until the Delta-SV was < 10%, according to French guidelines^3^ (Fig. 2).

The following demographic data were collected: age, gender, body mass index, medical background, American Society of Anaesthesiology Physical Status score (ASA), and type of surgery. The following ventilatory and hemodynamic data were collected at baseline and after each fluid challenge: plateau pressure, PEEP, minute ventilation, heart rate (HR), mean arterial pressure (MAP), pulsed oxygen saturation (SpO_2_), and cardiac index. The following data were collected at the end of the intervention: number of patients treated with norepinephrine, total intraoperative dose of norepinephrine (µg kg^-1^), total intraoperative volume of fluid administered (ml kg ^- 1^), arterial oxygen partial pressure to fractional inspired oxygen ratio (PaO_2_/FiO_2_) (mmHg), brain natriuretic peptide level, duration of the operation, length of hospitalization, and postoperative pulmonary complications.

The primary outcome was the fluid responsiveness status as assessed by Delta-SV. The secondary outcomes were postoperative pulmonary complications that were prospectively recorded 14 days after inclusion, and then classified (according to the European Perioperative Clinical Outcome definition)^19^.

The data analysis will consist of 3 parts:

1. Assessment of fluid unresponsiveness of Delta-B-line after the first fluid challenge.
2. Assessment of fluid unresponsiveness of Delta-B-line considering all fluid challenges.
3. Association between final Delta-B-line and pulmonary complications.

### Statistical analysis

A data analysis and statistical plan was written and posted on a publicly accessible server [Clinicaltrials.gov (NCT03502460)] before data were accessed. A sample of 200 patients was calculated to be sufficient to demonstrate that Delta-B-line could diagnose fluid unresponsiveness with an area under the curve > 0.70, under the assumption of an expected sensitivity of 80%, a power of 80%, and an alpha risk of 0.05.

The normality of the distribution of variables was verified using an Agostino-Pearson test. The data were expressed as the means ± standard deviation (SD), medians [interquartile interval (IQR)], or numbers (proportion in %), depending on the case. The Student t test, Mann–Whitney test, chi-squared test, or Fischer test were used to compare quantitative or qualitative variables between responding and non-responding patients and between patients developing complications and those who did not. The paired t test and Wilcoxon test were used to compare the variables before and after a 250-ml fluid challenge. Multivariable logistic regression was used to analyze the association between the occurrence of postoperative pulmonary complications and significant determinants in univariate analysis with a p-value < 0.05 and interactions between the centre and each variable. Diagnostic values for fluid unresponsiveness or postoperative pulmonary complications were assessed using a receiver operating characteristic curve and calculating the area under the curve. The best threshold was defined as that which provided the highest Youden index. Correlations between the Delta-SV and the Delta-B-line were tested using Spearman’s correlation rank analysis. Changes of the Delta-SV according of Delta-B-line were compared using one-way analysis of variance (ANOVA) with Tukey’s post hoc test. Differences with a p-value < 0.05 were considered statistically significant. Interobserver agreement between the two experts concerning qualitative ultrasound signs (signs present or absent) was evaluated using a Kappa concordance coefficient and agreement on quantitative evaluations (number of B-lines detected at the upper and lower sites) using an intraclass correlation coefficient.

MedCalc Statistical Software version 19.7 (MedCalc Software Ltd, Ostend, Belgium; https://www.medcalc.org; 2021) was used for all statistical analyses.

## Results

### Patients

**Figure 3:**
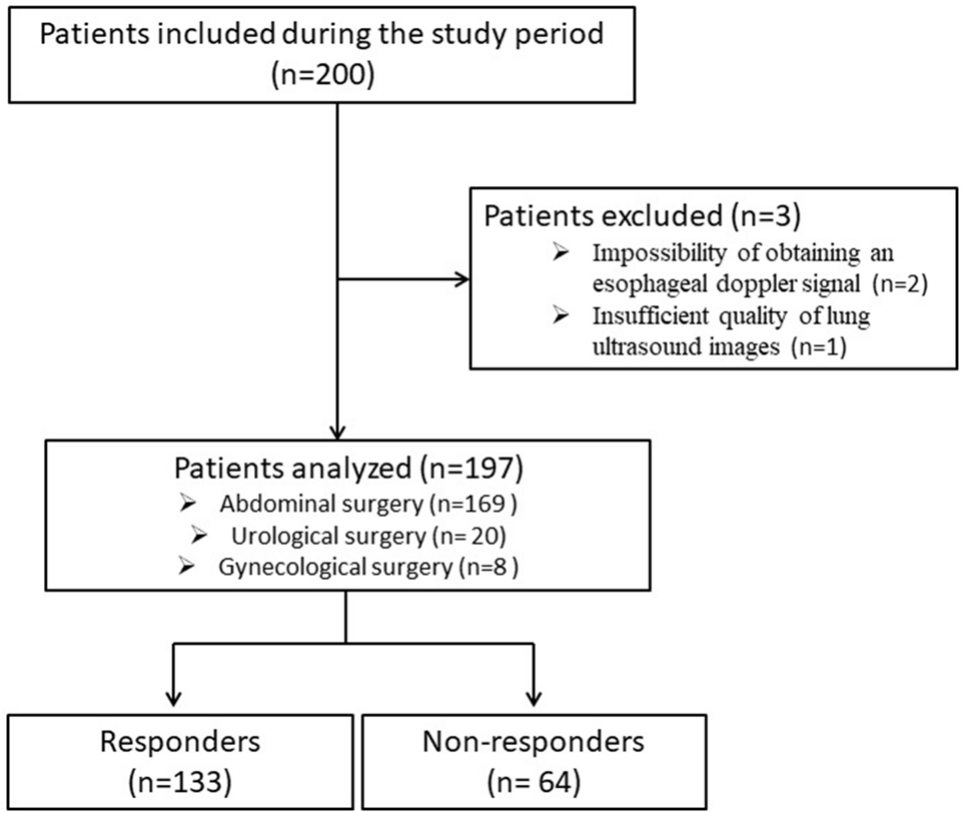
Flow chart of the study.

Of the 1196 eligible patients during the study period, 996 did not meet the enrolment criteria (593 without cardiac output monitoring, 318 with thoracic procedures, 39 with chronic interstitial lung disease, 38 with ARDS and eight refused to participate). 200 patients undergoing digestive, urological, or gynecological surgery who required intraoperative hemodynamic optimization by fluid challenge titration were finally included. Two cases were excluded because of the impossibility of obtaining an esophageal Doppler monitoring signal and one because of local interference that impeded the obtention of lung ultrasound images of sufficient quality (Fig. 3). The median [IQR] age was 62 [47–70] years, the median [IQR] ASA score was 2 [2–3]. There were 190 patients included in the University Hospital Center and 10 patients included in the General Hospital Center. There were no statistically significant centre effects, according to the results of a multivariate logistic regression adjusted for the interaction between the centre and each variable (p > 0.05).

### Assessment of fluid unresponsiveness after the first fluid challenge

The number of responding patients after the first fluid challenge (250 ml of crystalloids) was 133 (67%) and 64 were non-responders (33%). There was a significant decrease in HR and a significant increase in cardiac index in responding patients (p < 0.01). The number of B-lines increased in responders and non-responders after this first fluid challenge (p < 0.01) (Table 1). Delta-B-line was significantly higher in non-responders than responders (4 [2–8] *vs* 1 [0–3], p < 0.01). Delta-B-line was able to diagnose fluid non-responders with an area under the curve of 0.74 (95% CI 0.67 to 0.80, p < 0.01). The best threshold for Delta-B-line was two (sensitivity of 80%, specificity of 57%, positive likelihood ratio of 1.86 and negative likelihood ratio of 0.35). No patient received vasopressor before the first fluid challenge.

**Table 1:** Comparison of baseline characteristics, hemodynamic, echography, and ventilation parameters, at baseline and after the first fluid challenge of 250 ml, between responding and non-responding patients.

Characteristics	Responders (n = 133)	Non-responders (n = 64)	p-values
**Age; years**	61 [47–70]	62 [48–71]	0.88
**Female sex (%)**	59 (44%)	26 (40%)	0.28
**ASA-PS score**	2 [2–3]	2 [2–3]	0.25
**BMI; kg m**^**−2**^	25 [22–29]	27 [22–31]	0.29
**Medical background (%)**			
Arterial hypertension	58 (44%)	23 (36%)	0.26
Myocardial infarction	7 (5%)	5 (8%)	0.41
Cardiac dysfunction	2 (2%)	0	0.26
OSAS	10 (8%)	4 (6%)	0.25
Smoking	23 (17%)	8 (13%)	0.47
Arteritis	4 (3%)	0	0.16
Stroke	8 (6%)	3 (5%)	0.78
Diabetes	23 (17%)	12 (19%)	0.73
Dyslipidemia	28 (21%)	11 (17%)	0.50
Chronic renal disease	7 (5%)	4 (6%)	0.77
**HR; min**^**−1**^			
Baseline	69 [61–81]	73 [63–81]	0.61
250 ml	66 [58–80] *	71 [68–80]	**0.04**
**MAP; mmHg**			
Baseline	70 [64–78]	75 [65–82]	0.10
250 ml	69 [63–79]	75 [68–83]	**< 0.01**
**Cardiac index; l min**^**−1**^** m**^**−2**^			
Baseline	2.5 [2.0–3.2]	2.8 [2.3–3.3]	0.08
250 ml	2.9 [2.3–3.5]*	2.7 [2.2–3.5]	0.54
**SpO**_**2**_**; %**			
Baseline	99 ± 1	98 ± 2	**0.03**
250 ml	99 ± 1	98 ± 2	**< 0.01**
**Total B-lines**			
Baseline	0 [0–1]	0 [0–4]	**< 0.01**
250 ml	1 [0–3]*	4 [2–7]*	**< 0.01**
**Minute ventilation; l min**^**−1**^	6.3 [5.6–7.1]	6.4 [6.0–7.1]	0.30
**PEEP; cmH**_**2**_**O**	5 [5–6]	5 [5–6]	0.76
**Plateau pressure; cmH**_**2**_**O**	17 [15–19]	17 [15–19]	0.98

Significant values are in bold.

Values are means ± SD, numbers (proportion) or median [IQR].

*ASA-PS* American Society of Anesthesiology-Physical status, *BMI* body mass index, *HR* heart rate, *MAP* mean arterial pressure, *OSAS* obstructive sleep apnea syndrome, *SpO*_*2*_ pulsed oxygen saturation, *PEEP* positive end-expiratory pressure. The p-value refers to the intergroup comparison. *p < 0.05 for the intragroup effect.

### Assessment of fluid unresponsiveness considering all fluid challenges

**Figure 4:**
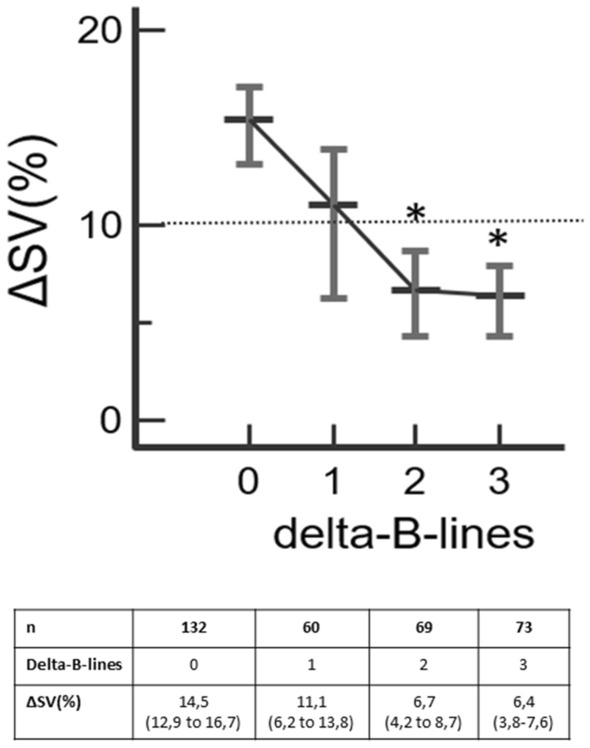
Analysis of the Delta-SV value according to the Delta-B-line. n = the number of datapoints for each value of the Delta-B-line. *p < 0.05 for the comparison to the Delta-SV for 0 Delta-B-line. Figure generated from MedCalc Statistical Software version 19.7 (MedCalc Software Ltd, Ostend, Belgium; https://www.medcalc.org; 2021).

When considering the population as a whole, 614 fluid challenges were performed, and the median total volume of fluid administered was 750 [500–1000] ml per patient. The Delta-B-line significantly negatively correlated with the value of the Delta-SV (rho = − 0.25, p < 0.01). When considering all fluid challenges administered, the Delta-SV values decrease significantly and below the threshold of 10% after the Delta-B-line ≥ 2 compared to Delta-B-line = 0 (Delta-SV_Delta-B-line=2_ = 6.7% 95% CI (4.2 to 8.7) and Delta-SV_Delta-B-line=3_ = 6.4% 95% CI (3.8 to 7.6) vs Delta-SV_Delta-B-line=0_ = 14.5% 95% CI (12.9 to 16.7); p < 0.05) (Fig. 4).

### Prediction of postoperative pulmonary complications

Sixteen (8%) patients developed postoperative pulmonary complications: eight (50%) developed a respiratory infection, four (25%) respiratory failure, and four (25%) aspiration pneumonitis. The final Delta-B-line was significantly higher for patients with pulmonary complications than those without (7 [5–12] *vs* 4 [2–7], p < 0.01) (Table 2). The final Delta-B-line was able to predict postoperative pulmonary complications with an area under the curve of 0.74 (95% CI 0.67 to 0.80, p < 0.01). The best threshold was five for the final Delta-B-line (sensitivity of 80%, specificity of 57%, positive likelihood ratio of 1.86 and negative likelihood ratio of 0.35).

**Table 2:** Comparison of baseline characteristics, echography, intraoperative, and postoperative parameters as a function of the occurrence of postoperative pulmonary complications.

Characteristics	Non-pulmonary complications (n = 181)	Pulmonary complication (n = 16)	p-value
**Age ; years**	61 [47–70]	66 [61–72]	**0.03**
**ASA-PS score**	2 [2–3]	2 [2–3]	0.26
**Medical background; %**			
Arterial hypertension	70 (39%)	11 (69%)	**0.02**
Myocardial infarction	11 (6%)	1 (6%)	1.00
Cardiac dysfunction	1 (0.6%)	1(6%)	0.05
OSAS	12 (7%)	2 (13%)	0.38
Smoking	31 (17%)	0 (0%)	0.07
Arteritis	2 (1%)	2 (13%)	**< 0.01**
Stroke	9 (5%)	2 (13%)	0.19
Diabetes	31 (17%)	4 (25%)	0.42
Dyslipidemia	35 (19%)	4 (25%)	0.26
Chronic renal disease	10 (6%)	1 (6%)	1.00
**Sugery; %**			
Digestive	157 (87%)	15 (94%)	0.42
Urological	14 (8%)	1 (6%)	0.78
Gynecological	10 (5%)	0 (0%)	0.36
**Final cardiac index; l min**^**−1**^** m**^**−2**^	3.1 [2.5–3.7]	3.4 [2.4–3.8]	0.69
**Final delta-B-line**	4 [2–7]	7 [5–12]	**< 0.01**
**Norepinephrine use; %**	60 (33%)	9 (56%)	0.07
**Norepinephrine dose; µg kg**^**−1**^	0.00 [0.00–0.04]	0.05 [0.00–0.08]	0.11
**Total fluid administered; ml kg**^**−1**^	9.4 [5.7–16.7]	8.4 [6.7–13.2]	0.67
**Total blood loss; ml kg**^**−1**^	4.9 [2.5–7.4]	6.4 [4.5–8.7]	0.66
**Intraoperative duration; min**	120 [60–210]	200 [130–240]	**0.01**
**Post-operative PaO**_**2**_**/FiO**_**2**_**; mmHg**	343 [273–419]	290 [173–402]	0.09
**Postoperative BNP; ng ml**^**−1**^	34 [16–92]	37 [13–120]	1.00
**Length of stay; days**	4 [2–8]	18 [13–36]	**< 0.01**

Significant values are in bold.

Values are means ± SD, numbers (proportion) or median [IQR].

*Digestive surgery* duodenopancreatectomy, hepatectomy, colectomy, gastrectomy, oesophagectomy, intraperitoneal hyperthermic chemotherapy. Urological surgery: cystectomy, nephrectomy, *Gynecological surgery* hysterectomy and ovariectomy, *OSAS* obstructive sleep apnea syndrome, *ASA-PS* American Society of Anesthesiology-Physical status, *PaO*_*2*_*/FiO*_*2*_ arterial oxygen partial pressure to fractional inspired oxygen ratio.

After multivariable logistic regression analysis, the final Delta-B-line was the only factor associated with postoperative pulmonary complications (OR = 1.22; 95% CI 1.06–1.41, p < 0.01) (Table 3).

**Table 3:** Univariable and multivariable logistic regression analysis for postoperative pulmonary complications.

Variables	Univariable analysis		Multivariable analysis	
	OR (CI 95%)	p-value	OR (CI 95%)	p-value
Age	1.04 (1.01–1.09)	**0.029**	1.05 (0.99–1.10)	0.081
Arterial hypertension	3.49 (1.21–11.46)	**0.026**	2.99 (0.97–9.48)	0.076
Arteritis	12.79 (1.45–113.40)	**0.014**	10.47 (0.99–106.23)	0.059
Delta-B-line	1.21 (1.06–1.37)	**0.003**	1.22 (1.06–1.41)	**0.007**
Intraoperative duration	1.00 (1.00–1.01)	**0.028**	1.01 (0.99–1.01)	0.069

Significant values are in bold.

Values are odds ratio (OR) with 95% CI.

Interobserver agreement was good, with a Kappa concordance coefficient of 0.87; 95% CI 0.67 to 0.99. The intraclass correlation coefficient for the agreement on quantitative evaluations was 0.90 (95% CI 0.80–0.96).

## Discussion

Our study suggests that Delta-B-line ≥ 2 at four zones can diagnose fluid unresponsiveness in patients undergoing abdominal surgery. Our study also found that the final Delta-B-line was associated with the occurrence of postoperative pulmonary complications.

B-lines result from an ultrasound reverberation phenomenon on subpleural inter-lobular septa and may reflect interstitial edema^12^. An increase in interstitial fluid may be caused by two phenomena. (1) An increase in hydrostatic pressures between the capillary and lung interstitium. A predominance of A-lines may predict low pulmonary artery occlusion pressure (PAOP) with good specificity, whereas a predominance of anterior B-lines may predict that the underlying lung is "overloaded"^20^. Thus, B-lines can be considered as a sign of high cardiac filling pressure^21^. Moreover, anaesthesia-induced vasodilatation induces decreased clearance and an increase in the half-life of infused fluids in healthy patients^22^. This could promote the slower turnover of crystalloids and the accumulation of fluid in the interstitium^23^. (2) Changes in capillary permeability. Some authors advocated an absence of correlation between the number of B-lines and an increase in the PAOP^24^. This can be explained by deterioration of the alveolar-capillary membrane^25^. Note that our population had “healthy” lungs. In the same population, it has been shown that a single bolus of crystalloids can cause interstitial pulmonary edema associated with a decrease in the angiopoietin-1/angiopoietin-2 ratio, suggesting a proinflammatory effect^26^. Still in these patients, hypervolemia could increase the release of atrial natriuretic peptide and cause enhanced shedding of the endothelial glycocalyx. This deterioration has been shown to cause the shifting of fluids into the interstitial space^27^.

The threshold for diagnosing fluid unresponsiveness may seem low (the appearance of a total of two B-lines in four zones), whereas current knowledge indicates that the number of B-lines must be ≥ 3 for diagnosing pulmonary edema^11^. We can explain our low threshold by the fact that the anaesthetist in charge of the patient was present to detect interstitial edema at a very early stage, whereas patients with acute pulmonary edema are explored at a more advanced stage. Thus, excess interstitial fluid may invade one or two sub-pleural interlobular septa, which is detected in near real-time during fluid challenge, leaving the other septa empty. A previous study showed that pre-lung rockets (two B-lines) may indicate an early stage of pulmonary edema^5^.

Finally, we found that the final Delta-B-line can predict the occurrence of postoperative pulmonary complications. Avoid fluid overload by using indexes guiding vascular filling have resulted in a significant reduction in pulmonary complications^28,29^. Studies have found that vascular filling in ICU patients with ARDS causes deterioration of lung aeration, visible by lung ultrasound. Such an excess of extravascular lung water has been shown to correlate with higher mortality^30^. In addition, the postoperative appearance of new B-lines following thoracic surgery has been shown to correlate with the development of postoperative pulmonary complications^31^. Moreover, it has been shown that the detection of B-lines in chronic hemodialysis patients is an independent factor of mortality and cardiovascular complications^32^. In our study, the median postoperative PaO_2_/FIO_2_ ratio for patients with complications was < 300 mmHg, which corresponds to “mild” ARDS according to the «Berlin definition», despite the absence of a significant difference relative to patients without complications^33^.

The assessment of perioperative hemodynamic is challenging and, thus, several tools are used. In addition, the accuracy of cardiac output monitors and the preload dependency index is still only modest^34^. Each monitor and index has its own advantages and limitations^35^. Finally, the adhesion and the weak compliance with the management protocol based on the monitoring tools of the SV currently, demonstrates the need to find indices based on tools already well used by the practitioners^36^. Lung ultrasound can therefore potentially be routinely used in the operating theatre to avoid fluid overload and thus reduce the occurrence of postoperative pulmonary complications. Lung ultrasound has several advantages. It is non-irradiating, noninvasive, and has a fast learning curve^37^. It is also worth remembering that B-lines are usually absent in euvolaemic patients and appear before clinical symptoms or signs of fluid overload^38^. Thus, it can allow continuous monitoring of B lines as soon as they appear, as well as dynamic reassessments of a patient's volume status. It is also a simple tool which the use is facilitated today by the development of portable devices. Moreover, it should be pointed out also that the ultrasound is cheap and is now available in many operating rooms. The interobserver agreement in this study was good, close to that of other studies^39^.

Our study had the following limitations. The first was the interpretation of B-lines that appear during surgery. Atelectasis occurring during the intervention was prevented by performing recruitment maneuvers. We were forced to remove patients with a suspected or proven acute lung disease or chronic interstitial lung disease to limit the occurrence of false positives and to avoid making it difficult to account of new B-lines appeared in case of many initial B-lines. However, in the case of our target population of elective surgery patients (excluding emergency surgery), trace patterns of incipiens pneumonia were a possible cause, although probably rare and anyway hard to prove currently.

Second, we saw that the accuracy of lung ultrasound was good but not perfect. We expected such results, which indicated that either lung ultrasound was not perfect, or that the gold standard was not perfect too. Comparing a new test to an imperfect gold standard might unavoidably generate less than perfect results. Note that the esophageal Doppler monitoring probe, which is one of the cardiac output monitoring devices frequently used in the operating theatre, has its own limitations, including spatial instability in the esophagus and accuracy of measurement of the cardiac output^34,40^. The results of this study should be understood with this critical point in mind.

Thirdly, we must keep in mind that a volume responsive patient may be intolerant to fluids, so while a Delta B line should definitely represent a fluid stop point, it may not represent volume unresponsiveness, which likely explains lung ultrasound being "imperfect" as a fluid responsiveness tool, which it isn't inherently. It is a marker of fluid tolerance and further studies should be carried out taking into account this endpoint.

Finally, our results are based on an optimization by the variation of the stroke volume and not by the variation of the number of B lines in ultrasound because this method is not yet recognized. A randomized and multicenter study project will be set up to show the superiority in terms of postoperative complications of a fluid titration strategy based on delta-B-line versus stroke volume monitoring.

## Conclusion

This study showed that Delta-B-line of two or more detected in four lung ultrasound zones can be considered to be a marker of preload unresponsiveness after fluid challenge in abdominal surgery (Supplmentary Information).

## Supplementary Information

Supplementary Information.

## Data Availability

The datasets used and/or analysed during the current study are available from the corresponding author on reasonable request.

## Code availability

Code are available from the corresponding author on reasonable request.
